# Enhancing Dentists’ Resilience and Occupational Sustainability Through Physical Activity: A Systematic Review in the Post-Pandemic Context

**DOI:** 10.3390/healthcare13161985

**Published:** 2025-08-13

**Authors:** Theodora Kalogerakou, Maria Antoniadou

**Affiliations:** 1 Department of Dentistry, School of Health Sciences, National and Kapodistrian University of Athens, 11527 Athens, Greece; tkalogerakou@hotmail.com; 2 Certified Systemic Analyst Program, University of Piraeus, 18534 Piraeus, Greece

**Keywords:** dentists, occupational health, physical activity, workplace wellness, stress management, mental health, resilience, sustainability, ergonomic support, well-being

## Abstract

Background: Dental professionals face high levels of occupational stress, which intensified during the COVID-19 pandemic, contributing to increased burnout, diminished well-being, and signs of accelerated biological aging. This systematic review explores the role of physical activity as a protective factor for mental and physical health, with a focus on promoting resilience and long-term occupational sustainability in a post-pandemic volatile, uncertain, complex, and ambiguous (VUCA) environment. Methods: A systematic review was conducted in accordance with PRISMA 2020 guidelines. Studies published between 2000 and 2024 were identified through PubMed, Scopus, and the Cochrane Library using MeSH terms related to dentistry, physical activity, stress management, and occupational health. Of 850 records screened, 28 studies were included: 24 cross-sectional, 2 systematic reviews, 1 retrospective, and 1 case–control study. Inclusion and quality appraisal followed standardized criteria. Results: The included studies consistently showed that physical activity was associated with reduced burnout, improved psychological well-being, enhanced postural function, and potential markers of slowed biological aging in dental professionals. Several studies reported moderate-to-strong associations, with effect sizes ranging from small improvements in perceived stress scores to substantial reductions in MSD prevalence. Interventions combining exercise with ergonomic education, stress management, and institutional support demonstrated the stronger and more consistent benefits for professional sustainability. Conclusions: Physical activity, when integrated into comprehensive workplace wellness frameworks, significantly enhances the mental and physical resilience of dental professionals. Embedding movement, ergonomics, and psychosocial support into practice environments offers a promising strategy for safeguarding long-term workforce sustainability and improving public health outcomes.

## 1. Introduction

Dentistry is widely recognized as one of the most physically and mentally demanding healthcare professions [1]. Dental practitioners are required to maintain high levels of technical proficiency, precision, and sustained concentration, often while performing repetitive procedures in confined and ergonomically challenging environments [2,3]. These demands pose a significant problem for practitioners’ long-term well-being and career sustainability, which this review explicitly aims to address.

The physical demands of the profession are compounded by psychological stressors, such as managing patient expectations, minimizing procedural errors, and achieving optimal clinical outcomes, factors that can significantly affect both patient satisfaction and practitioners’ well-being [4]. Even minor procedural mistakes can have serious implications, thereby intensifying the psychological pressure on practitioners [5,6]. Here, psychological resilience, the capacity to adapt to stress and recover from adversity, becomes crucial to coping with these challenges [7]. In addition, long working hours, continuous education requirements, and the obligation to provide high-quality care under time constraints further augment mental fatigue and emotional exhaustion [8,9]. At the same time, the prevalence of musculoskeletal disorders (MSDs) among dentists remains alarmingly high [10]. These conditions have been linked to sustained awkward postures, repetitive hand movements, and prolonged static positioning during clinical procedures [5,10]. Common issues include back and neck pain, shoulder stiffness, and carpal tunnel syndrome, which significantly impair quality of life and may even lead to early retirement from clinical practice [4]. This illustrates how physical, psychological, and organizational factors interact to undermine occupational health and sustainability in dentistry [4,5,6,7].

In response to these occupational hazards, increasing attention has turned to modifiable protective factors, particularly regular physical activity, as means of promoting resilience and occupational sustainability among healthcare professionals [11]. In this review, occupational sustainability refers to the ability to maintain health, performance, and engagement in the profession over time while reducing the risks of premature exit from clinical practice [12,13]. Physical activity is then associated with numerous health benefits, including reduced stress, enhanced cognitive performance, and improved mood regulation, factors essential for managing the high cognitive and emotional demands of dentistry [6]. In addition, strengthening core muscles and enhancing posture through regular exercise may reduce the ergonomic strain faced by dental professionals [8]. Aerobic activities such as running and swimming are also known to stimulate endorphin release, which contributes to emotional well-being in high-stress professions [14]. Furthermore, regular physical activity contributes to better sleep quality, which is critical for maintaining attention, energy levels, and overall performance during long clinical shifts [6,8,9,15,16]. Beyond general wellness, physical activity has been shown to positively influence biological aging by regulating circadian rhythms and decreasing oxidative stress markers [17]. While not the primary focus of this article, the topic of biological aging, defined as the accumulation of cellular and molecular damage over time, which may be accelerated by chronic stress and physical strain but potentially controlled through exercise, will be briefly addressed with the broader literature [18,19].

Dentists are particularly susceptible to accelerated cellular aging due to chronic inflammation and prolonged physical stress [18], but these effects may be reduced through exercise, which promotes musculoskeletal health, stabilizes biological rhythms, and supports psychological resilience [19,20]. Although the benefits are well-documented, many dentists struggle to incorporate regular physical activity into their routines due to fatigue, workload, and lack of institutional support [21]. These barriers were augmented during the COVID-19 pandemic, which increased workload, introduced stricter clinical protocols, disrupted routines, and heightened mental strain across the profession [22,23]. Post-pandemic findings report the need for strategies that enhance psychological resilience and reduce burnout, with physical activity identified as a critical component for achieving long-term professional sustainability [6,24,25,26].

Despite growing evidence, a significant gap remains in the literature on how physical activity specifically supports the occupational health of dental professionals. Many existing reviews focus on general healthcare workers, rely on pre-pandemic data that may not reflect current dental practice realities, or lack dentist-specific data altogether [27,28,29,30]. Furthermore, few interventions have been designed to address the unique ergonomic and psychological demands faced by dentists in contemporary settings [31]. This review aims to fill this gap by synthesizing recent evidence on how physical activity enhances both physical and mental resilience among dentists, particularly in the post-pandemic context, and by identifying practical, evidence-based strategies to integrate exercise into clinical routines and promote healthy aging, resilience, and career sustainability.

## 2. Materials and Methods

In this systematic review, we followed guidelines provided by PROSPERO (https://www.crd.york.ac.uk/prospero/, accessed on 20 January 2025) and informed by the systematic review protocol registered under ID CRD42024523797. The methodology involves a thorough search strategy utilizing databases such as PubMed, Scopus, and the Cochrane Library, focusing on quantitative and experimental studies to understand how the pandemic has reshaped the health and work–life conditions of dental professionals. The search was conducted using Boolean operators (e.g., “AND”, “OR”) and included combinations of keywords such as “dentists”, “physical activity”, “mental health”, “aging”, “circadian rhythm”, “career sustainability”, and “anti-aging”. Among these, “dentists”, “physical activity”, and “mental health” were explicitly used as database search terms, while “aging”, “circadian rhythm”, “career sustainability”, and “anti-aging” emerged during full-text analysis and synthesis and were not part of the formal search query. These keywords were selected additionally to capture studies that explore the multifaceted effects of physical activity on both physical and psychological aspects of dental professionals’ well-being, including its potential role in regulating biological rhythms, supporting longevity, and promoting professional resilience. Filters were applied to limit results to human studies, the English language, and publication years from 2000 to March 2024.

The databases used in this study were selected due to their broad coverage of biomedical, public health, and psychological research, ensuring access to the peer-reviewed, high-quality literature relevant to the intersection of physical activity and occupational health [31,32,33]. The extended publication window (2000–2024) was intentionally chosen to allow for the inclusion of foundational and pre-pandemic research, enabling the comparison of patterns and trends before and after COVID-19. Although our primary interest is in post-pandemic outcomes, earlier studies provided essential baseline data for context and highlighted pre-existing risks and resilience factors in dental professionals. This helped us contextualize post-pandemic findings within a broader historical framework. A formal comparative analysis between pre- and post-pandemic studies was not performed, as differences in study designs, outcomes measured, and populations studied introduced substantial heterogeneity, making direct statistical comparison inappropriate. Overall, this approach aimed to identify a broad range of relevant studies and minimize the exclusion of potentially significant findings [34]. Additionally, quality assessment, data extraction, and statistical analysis have been conducted, following standardized protocols such as the Newcastle–Ottawa scale and SPSS tools [35]. We also conducted our search following the PRISMA guidelines (Preferred Reporting Items for Systematic Reviews and Meta-Analyses) (https://www.prisma-statement.org/, accessed on 23 March 2025) (Table S1).

Furthermore, inclusion and exclusion criteria were applied to select studies for this review. Studies were eligible if published between 2000 and March 2024 and written in English. We limited studies to those in English to allow accessibility to the review team. To ensure a comprehensive dataset, the review included cross-sectional studies (observational studies that analyze data from a population at a specific point in time), case–control studies (studies comparing subjects with a specific condition to those without), and experimental studies (research involving intervention and control groups). We excluded prospective and cohort studies because we aimed to synthesize observational snapshots and controlled interventions rather than long-term follow-up data. Further, although prospective and cohort studies are valuable for understanding long-term associations and causality, they were excluded from this review due to heterogeneity in design and limited availability in the dental field. We acknowledge that this exclusion may limit the strength of the evidence and the ability to conclude long-term outcomes. However, our emphasis on cross-sectional and interventional studies aligns with the goal of identifying actionable, short-term strategies to address musculoskeletal disorders and promote wellness in dental professionals. This focus additionally allowed for a more uniform comparative analysis across studies [36]. Also, studies conducted in mixed healthcare populations were excluded if they did not report results for dentists separately, to maintain focus on the target population. Overall, exclusion criteria were applied to studies that did not focus on dentists or did not consider physical activity as a key factor.

The review process included a thorough assessment of the methodological quality of the selected studies to ensure they met good scientific standards, enhancing the validity of the findings [37]. To evaluate the quality of the included studies, we applied the Newcastle–Ottawa Scale (NOS) (https://www.ohri.ca/programs/clinical_epidemiology/oxford.asp, accessed on 27 March 2025) for observational studies and the JBI Critical Appraisal Tools (https://jbi.global/critical-appraisal-tools, accessed on 27 March 2025) (Table S2). The resulting quality scores informed our interpretation of the evidence, with higher-quality studies given greater weight in our synthesis of findings. Title and abstract screening, full-text selection, and data extraction were performed independently by the two authors. Quality assessment was also performed independently by these reviewers, and disagreements were resolved by discussion or consensus. Inter-rater reliability was substantial, confirming the consistency of the classification process.

Regarding statistical analysis, descriptive statistics (e.g., frequencies, percentages) were calculated to characterize the included studies and their main outcomes using SPSS (IBM SPSS Statistics, v31 powered). Outcomes analyzed included prevalence and types of musculoskeletal disorders, stress and burnout indicators, sleep quality, resilience scores, and measures of biological aging where reported.

## 3. Results

**Figure 1:**
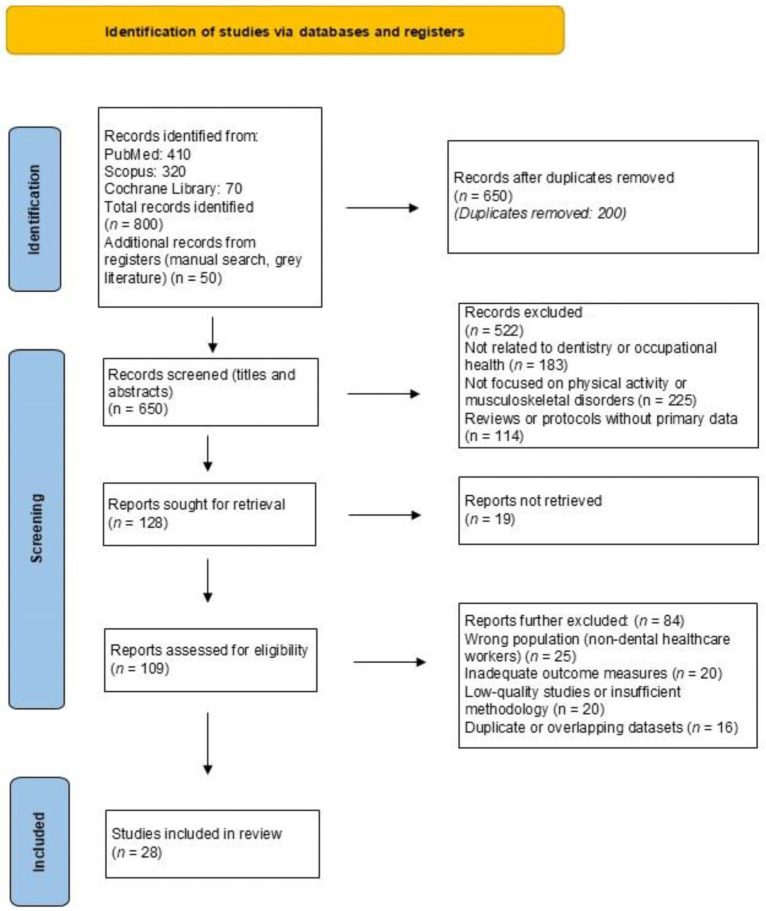
Flowchart of the search strategy results.

In Figure 1, the PRISMA flow chart of the study is presented. A total of 800 records were initially identified through electronic databases, PubMed (*n* = 410), Scopus (*n* = 320), and the Cochrane Library (*n* = 70). An additional 50 records were identified through manual searches, the gray literature, and other registers, bringing the total number of identified records to 850. After removing 200 duplicates, 650 records remained for screening at the title and abstract level. Of these, 522 records were excluded for the following reasons: irrelevance to dentistry or occupational health (*n* = 183), not focusing on physical activity or musculoskeletal disorders (*n* = 225), and being review articles or protocols without primary data (*n* = 114). This left 128 records for full-text retrieval. Of these, 19 could not be retrieved. The remaining 109 full-text articles were assessed for eligibility. A total of 84 articles were further excluded for the following reasons: wrong population (non-dental healthcare workers) (*n* = 25), inadequate outcome measures (*n* = 20), low-quality studies or insufficient methodology (*n* = 20), and duplicate or overlapping datasets (*n* = 16). Ultimately, 28 studies were included in the final review.

The twenty-eight studies represented a broad range of geographical regions and provided diverse insights into occupational health among dentists. Details on the study characteristics can be seen in Table 1.

The methodological quality and reliability of the included studies were appraised using standardized criteria, and the detailed assessment is presented in Table S1.

### 3.1. Trends in Research on Physical Activity and Dental Health

**Figure 2:**
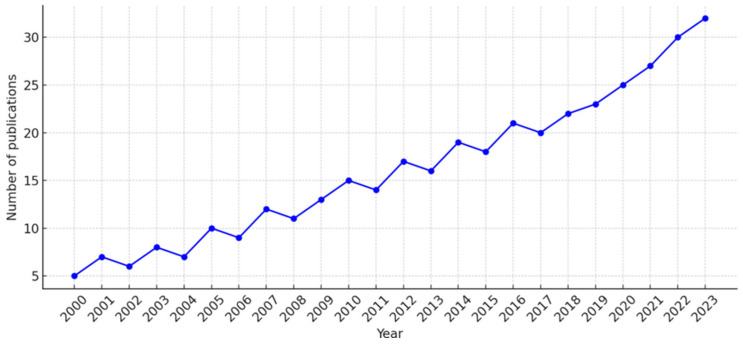
Trend chart showing the articles published in the PubMed database during the period 2000–2024 with the search phrase “physical activity of dentists” in the title and/or abstract.

A notable trend in the literature is the predominant focus on musculoskeletal disorders (MSDs), often overshadowing mental health and job satisfaction in dental professionals. As shown in Figure 2, research on the impact of physical activity in dentistry has steadily increased from 2000 to 2024. The early 2000s saw fewer than 10 publications per year, but output rose gradually after 2010, accelerating sharply post-2017, likely driven by heightened awareness during the COVID-19 pandemic [47,50]. Many recent studies emphasize not only the high prevalence of MSDs and the importance of ergonomics but also the psychological strain and burnout dentists face [10,52,53,54,55,56,57,58]. This growing body of evidence underlines the dual role of physical activity in preventing MSDs and enhancing emotional resilience and occupational well-being (Figure 2).

### 3.2. Geographic and Sectoral Distribution of Included Studies

The studies included in this review span a diverse range of countries, offering insights into occupational health among dental professionals globally. Three studies. each originated from Turkey and India (10% each), followed by two each from Italy, China, Pakistan, and Romania (6.7% each). Single studies (≈3.3% each) came from Finland, Afghanistan, Yemen, Portugal, Lebanon, Bangladesh, Bahrain, Canada, Kuwait, Iran, the UAE, South Africa, Germany, Poland, Greece, and Croatia. Most studies did not specify whether they were conducted in public, private, or mixed-sector settings.

### 3.3. Population and Sample Size

The sample sizes varied considerably across the studies included. They ranged from small (e.g., 50 participants in Javed et al., 2023) to large (e.g., 1500 dentists in Berdouses et al., 2020) [42,48], reflecting the variability in study scope and design. Larger-scale studies, such as the one conducted by Al-Huthaifi et al. (2023) in Yemen involving 310 dental professionals, offer stronger data compared to the one from Pakistan with a smaller sample [37,42]. Similarly, Gandolfi et al. (2021) in Italy surveyed 284 dental professionals, while Berdouses et al. (2020) conducted a nationwide survey in Greece with a substantial sample of 1500 dentists [10,48]. Both studies provide more reliable estimates regarding the prevalence of musculoskeletal disorders (MSDs) and other occupational health concerns.

### 3.4. Data Collection Methods

The majority of the included studies relied on self-administered questionnaires, a widely adopted and practical approach for assessing occupational health in dental professionals [5,6]. This method enabled efficient, large-scale data collection and provided both quantitative prevalence estimates and qualitative perceptions of musculoskeletal disorders (MSDs), workplace conditions, and health behaviors. Fourteen studies explicitly reported using questionnaires as their primary data source [4,7,10,30,37,38,39,40,41,42,48,49,50,57], while at least one used interviews to explore individual experiences in greater depth [7].

### 3.5. Statistical Analysis Methods

According to the studies, structured statistical analyses were consistently employed to examine associations between occupational factors (e.g., posture, workload, ergonomic practices) and health outcomes. Descriptive statistics and chi-square tests were commonly used to identify significant differences across groups, such as between genders, career stages, or working hours, with Macrì et al. (2023) [41] and Al-Huthaifi et al. (2023) [37] illustrating this well in their comparative analyses. Several studies, including Al-Emara et al. (2024) [39], applied correlation analyses and age-adjusted models to assess the impact of MSDs on work ability. More advanced regression techniques, particularly multivariate models, featured prominently to control confounding variables such as age, gender, years of practice, and ergonomic factors. Notably, Matur et al. (2023) [40] demonstrated how both ergonomic practices and duration of hand use were significant predictors of carpal tunnel syndrome after adjusting for these confounders.

### 3.6. Cultural Diversities

The reviewed studies offer a valuable cross-cultural perspective on how workplace practices, economic conditions, and healthcare infrastructure influence occupational health among dental professionals, emphasizing the need for context-specific preventive strategies. Across diverse settings, risks such as musculoskeletal disorders (MSDs), burnout, and work-related stress were consistently reported, though their prevalence and severity varied according to sociocultural, systemic, and individual factors.

More specifically, sociocultural and systemic factors strongly shaped occupational risks. Macrì et al. (2023) [41] identified differences in MSD prevalence between Italy and Peru, associated with gender, work hours, and physical activity, underscoring how cultural norms and institutional resources affect outcomes. Similarly, Al-Huthaifi et al. (2023) [37] highlighted how insufficient ergonomic education and lack of standardized protocols in Yemen contribute to high injury rates, while Al-Emara et al. (2024) [39] showed that even in high-income Finland, MSDs reduced work ability and increased disability risk, demonstrating that strong healthcare systems alone are insufficient to control occupational hazards.

Gender, experience, and lifestyle factors also emerged as consistent predictors of poor outcomes. Studies reported higher MSD prevalence and lower physical activity among women compared to men, reflecting gender disparities in risk and coping [10,42,52]. Gandolfi et al. (2021) [10] and others [42,52] linked MSDs to longer work hours and greater professional experience, particularly among women. Long working hours, low physical activity, and high stress were also strongly associated with MSDs and obesity in Indian practitioners [43], while Hashim & Al-Ali (2013) [54] extended these findings, suggesting widespread systemic health issues and unhealthy habits in UAE dentists. Although these patterns were broadly consistent, the magnitude of gender differences and unhealthy behaviors varied across contexts, likely reflecting differences in institutional support and sociocultural attitudes. Studies that applied multivariate models [10,43] strengthened the evidence for these associations, though self-reported data remains always a limitation.

Further, in lower-resource settings, stress and fatigue were particularly pronounced. Azimi et al. (2024) [4] in Afghanistan reported high fatigue and back pain, with dentists perceiving their profession as highly stressful. Similarly, Asaduzzaman et al. (2022) [36] observed pronounced ergonomic challenges among older practitioners in Bangladesh, suggesting cumulative exposure over time.

Finally, comparative studies illustrated the distinct burden of dental work compared to other occupations. Zhou et al. (2021) [46] found dentists in China experienced higher neck pain and lower pain thresholds than office workers, while Harris et al. (2020) [51] in Canada reported that 83% of hygienists experienced at least one MSD, with risk increasing with years of practice emphasizing the cumulative toll of long-term ergonomic strain.

### 3.7. Prevalence and Risk Factors of Musculoskeletal Disorders (MSDs)

Literature consistently reports a high prevalence of musculoskeletal disorders (MSDs) among dental professionals, particularly in the back, neck, shoulders, and wrists, caused by static postures, repetitive movements, and awkward working angles [37,39]. So common ergonomic risks include poor posture, insufficient breaks, high patient loads, and inadequate equipment [48,49,56,58]. For example, Greek and Croatian dentists reported high MSD rates linked to posture, noise, and stress [48,49], while similar patterns of neck, back, and lower limb pain were observed in South Africa and Poland, associated with prolonged static work and long careers [56,57,58]. We should also note that professional rank and lifestyle influenced outcomes: interns and students, who were more physically active, showed lower MSD rates than senior faculty [55].

### 3.8. Physical Activity as a Protective Factor

Physical activity emerges as a critical, modifiable factor in addressing both the physical and psychological demands of dental practice. Evidence suggests that it not only alleviates musculoskeletal strain but also enhances mental resilience and reduces burnout risk. For example, Azimi et al. (2024) [4] demonstrated that regular physical activity significantly reduced fatigue and improved mental well-being among dentists, supporting its role in managing the profession’s high physical and emotional demands. Similarly, other studies confirmed that physical activity, as part of broader coping strategies, effectively reduced stress, burnout, and secondary traumatic stress in high-pressure dental settings [59,60,61,62,63]. Beyond its psychological benefits, physical activity also protects against musculoskeletal disorders [64,65]. Matur et al. (2023) found that it reduced symptoms of carpal tunnel syndrome and reduced the ergonomic strain associated with repetitive hand movements and static postures characteristic of dental work [40].

### 3.9. The Role of Physical Activity in Regulating Sleep, Coping with Stress, and Building Resilience

Physical activity not only reduces stress but also supports circadian rhythm regulation and sleep quality, both crucial for sustaining health and preventing burnout. Kurtović et al. (2023) [9] emphasized the importance of balanced sleep and regular activity in enhancing resilience and reducing occupational burnout among dental professionals. Similarly, Singh et al. (2016) [66] recommended physical activity as part of a balanced lifestyle to counteract stress and maintain well-being. The COVID-19 pandemic further reported on this need. For example, Mekhemar et al. (2021) [26] and Szalai et al. (2021) [67] reported that physical activity, combined with mental health support, helped dentists manage heightened anxiety and burnout during the crisis. Finally, White et al. (2024) [68] highlighted that physical activity improves mental health not only directly but also indirectly by enhancing self-efficacy and social support, mechanisms particularly relevant for resilience in high-stress professions like dentistry [69].

### 3.10. Stress Management and Holistic Interventions in Dentistry

Dental professionals experience high levels of occupational stress and fatigue, largely driven by long working hours, financial pressures, and the demands of patient care and practice management [39,70]. Studies identify “long working hours” and post-work fatigue as the most significant stressors, with financial and patient-related pressures further compounding their impact on mental well-being and career satisfaction [39,59,70]. To address these challenges, psychological interventions such as cognitive-behavioral therapy (CBT), mindfulness-based stress reduction (MBSR), and relaxation training have been shown to enhance coping strategies, improve self-regulation, and reduce emotional reactivity in high-stress clinical environments [4,41,59,71]. Neumann et al. (2022) [72] further noted that higher physical fitness is associated with greater psychological resilience, mediated by self-efficacy. Complementary, physical and ergonomic strategies such as strengthening exercises, posture training, and workstation adjustments help maintain physical resilience and reduce the risk of musculoskeletal disorders (MSDs) [60,62,70,73]. Holistic approaches that integrate physical activity, mental health support, adequate rest, balanced nutrition, and regular breaks have been shown to reduce burnout, enhance job satisfaction, and cultivate healthier work environments [4,39,63].

### 3.11. Tailored Interventions and Cultural Approaches to Occupational Health

Studies highlight that effective occupational health interventions in dentistry vary across settings, reflecting both cultural and institutional differences. Some prioritize physical modifications, such as ergonomic chairs and optimized workstation layouts, to reduce MSDs [73], while others emphasize psychological support, including stress management programs and work–life balance adjustments, to address mental strain [4,71,74]. These differences derive from variations in study design, population, and cultural norms, which shape whether physical or mental health receives more focus [41,75].

Additionally, cultural context strongly influences intervention priorities. In some regions, workplace ergonomics is emphasized, while in others, mental health and work–life balance take precedence [59,75]. Consequently, a tailored approach that integrates ergonomic improvements with mental health support and aligns with regional practices appears most effective, enhancing job satisfaction, reducing burnout, and improving patient care outcomes [75].

### 3.12. The Importance of Education and Continuous Professional Development

Education and training are essential for reducing both physical and mental strain among dental professionals, forming the foundation for improved occupational health [61]. Targeted ergonomic education helps dentists maintain proper posture, use ergonomic tools effectively, and integrate these practices into daily routines, minimizing strain and promoting long-term health [4,37,41]. Further, a comprehensive approach combining ergonomic training, physical activity, and mental health support enhances career sustainability by equipping practitioners to manage stress and prevent burnout [70,71]. Workshops and professional development programs on ergonomics, posture, and stress management further promote healthy habits and awareness of body mechanics [76,77].

Addressing sedentary behavior is also critical. Scheepers et al. (2020) [78] linked inactivity to obesity and poor health outcomes, while wellness programs enhancing active lifestyles were shown to improve overall health and reduce chronic disease risks [39,79]. The World Health Organization (2022) [80] and subsequent studies [41,77,78,81] support workplace wellness initiatives, combining ergonomic training, structured activities, and mental health resources, as effective means of improving productivity, job satisfaction, and well-being.

### 3.13. Sensitivity Analysis

To assess the review’s conclusions, a sensitivity analysis was performed by sequentially altering key inclusion criteria (Table 2). The findings demonstrated that excluding studies with smaller sample sizes had a low to moderate impact on overall conclusions, with larger-scale research reinforcing previously identified trends. Limiting the analysis to studies employing standardized diagnostic tools showed minimal deviation from the main findings, suggesting strong methodological consistency. However, removing studies from low- and middle-income countries (LMICs) revealed a moderate to high impact, highlighting their role in emphasizing contextual and systemic dimensions of occupational health. Similarly, restricting the dataset to only those studies specifying the healthcare sector led to significant data reduction, resulting in weaker conclusions. Lastly, focusing solely on post-2020 publications caused a thematic shift, with more emphasis on mental health and pandemic-related stressors, while long-standing trends in musculoskeletal disorders were diluted. These results support the stability of core findings while reporting on the importance of inclusive and diverse study populations.

## 4. Discussion

Previous systematic reviews and meta-analyses have primarily focused on estimating the prevalence of musculoskeletal disorders (MSDs) among dental professionals, providing valuable epidemiological insights into how these conditions vary across regions and professional roles [43]. However, these studies often fail to integrate the physical, psychological, and behavioral dimensions of occupational health into a coherent framework. In the present review, we address this gap by adopting a holistic perspective, examining the connection between MSDs, psychological stress, burnout, biological aging, and postural strain. In doing so, the role of physical activity is highlighted as a modifiable protective factor, demonstrating how structured lifestyle interventions can enhance resilience, reduce long-term occupational risks, and support professional sustainability, especially relevant in the post-pandemic context of heightened psychosocial and clinical demands.

### 4.1. Practical Recommendations for Dental Professionals and Institutions

Building on the evidence reviewed, including emerging research on the biological and cellular effects of physical activity [82], several actionable and cost-effective strategies can support dental professionals and institutions in reducing musculoskeletal disorders (MSDs), enhancing well-being, and promoting sustainable, long-term careers. These recommendations operate at both individual and institutional levels, addressing not only ergonomic and behavioral interventions but also broader physiological resilience mechanisms, such as those related to cellular stress and neuroplasticity [81,82,83,84,85]. Importantly, they take into account barriers to implementation and variations across clinical settings, ensuring their relevance and feasibility in diverse dental practice environments.

At the individual level, dental practitioners are encouraged to integrate regular physical activity into their routines, as this has consistently been shown to reduce MSD symptoms and improve resilience [10,38,42,52]. Practical steps include performing brief stretching exercises between patients, incorporating aerobic activities outside of work, and strengthening core and postural muscles [4,39,40]. Practitioners should also prioritize ergonomic awareness by adjusting seating, operatory height, and light, and maintaining neutral postures during procedures [37,41,48]. Inexpensive aids, such as ergonomic chairs, properly angled loupes, and wrist supports, can contribute significantly to reducing strain [30,43,57].

In small private practices, where resources are limited, low-cost interventions like staff-led ergonomic workshops, educational posters, and reorganization of workspaces to support better posture can yield measurable benefits [36,45,54]. Encouraging a supportive culture where scheduled micro-breaks and open discussions about health are normalized can help overcome resistance derived from workload pressures or cultural norms [7,29,55,86].

Moreover, in larger institutional or public settings, leadership should play an active role in ensuring occupational health. This can include a designed plan which provides access to fitness facilities or arranging partnerships with local gyms [44,51], schedules mandatory rest breaks to prevent overuse injuries [39,46], and organizes formal training on ergonomics and stress management [41,56]. Institutional investments in adjustable chairs, operator stools, and improved lighting and focusing vision through loops are cost-effective when weighed against the costs of absenteeism and reduced productivity due to MSDs [48,50,53].

Finally, implementation barriers, such as lack of time, insufficient awareness, cultural resistance to self-care, and budget constraints, need to be addressed explicitly [37,42,52]. Framing wellness initiatives as essential to patient safety and professional performance rather than as optional can augment the uptake [10,43,49]. In lower-resource contexts, creative solutions such as shared ergonomic equipment, peer-led training sessions, and free or low-cost digital tools (e.g., mobile apps for guided exercises or posture monitoring) can bridge gaps [54,57,58]. Overall, the evidence suggests that early, small, and sustained changes in practice routines, supported by institutional policies and a positive workplace culture, are the most effective and cost-efficient way to reduce MSDs and enhance well-being [38,39,40,41,42,43,44,45,46,47,48,49,50,51,52,53,54,55,56,57,58,87]. Embedding these behaviors into the professional identity of dental practitioners cultivates long-term resilience and healthier careers [10].

### 4.2. Professional Identity and Role Modeling

From our investigation, it is derived that self-care is not only a personal health strategy but also an expression of professional identity and leadership in dentistry [88,89,90,91]. Dentists who prioritize physical activity, stress management, and ergonomic practices exemplify the values of health, responsibility, and sustainability central to modern healthcare [1,2,3,4,5]. Such behaviors serve as role modeling for colleagues, staff, and patients, reinforcing the notion that maintaining health is integral to professional excellence [5,92]. In clinical settings, where norms are transmitted through observation, dentists who incorporate wellness habits such as regular movement, good posture, and open stress management help shape expectations and cultivate a culture of well-being [4,5,93]. These practices strengthen team resilience, improve psychological safety, and align with emerging holistic care models that emphasize preventive health for both providers and patients [5,51,91,94,95,96]. Integrating personal wellness into daily conduct allows dental professionals to protect their health, and, in the meantime, they demonstrate leadership skills that prioritizes sustainable and competent care delivery [91,97].

Although not the primary focus of this article, it is important to acknowledge that, within the broader literature, recovery, particularly through adequate sleep and its interaction with physical activity and circadian regulation, plays a critical yet often underrecognized role in occupational health in dentistry [9,66,70,98,99,100,101,102,103,104,105]. Recent evidence highlights how structured physical activity can enhance sleep quality, support circadian alignment [103], and positively influence neurocognitive outcomes such as memory, executive function, and emotional regulation [99,100,102]. Poor sleep impairs cognitive performance, emotional regulation, immune function, and increases systemic inflammation, potentially undermining the benefits of exercise and ergonomic interventions [8,9,66]. Dentists’ intense schedules and irregular hours heighten their risk for chronic sleep disruption and insufficient recovery, which are strongly associated with burnout, higher MSD risk, and reduced clinical performance [4,70]. Insufficient recovery also compromises neuroplasticity, executive functioning, and metabolic regulation, key components of professional resilience [72]. Adopting sleep hygiene measures, such as minimizing late-night screen exposure, aligning work with circadian rhythms, and incorporating restorative breaks, can substantially improve recovery [9,84]. High-quality sleep even influences epigenetic mechanisms, such as upregulation of protective genes like S-Klotho, promoting systemic rejuvenation and reducing age-related decline [95,97,106,107,108]. Consequently, recovery should be recognized as a core pillar of occupational health and be integrated into workplace wellness initiatives alongside physical activity and ergonomic strategies to support long-term resilience and professional sustainability [93,109,110,111].

### 4.3. Contextualizing Interventions by Culture and Resources

Occupational health strategies in dentistry must account for local culture, resource constraints, and institutional norms [37]. Interventions effective in one region may fail elsewhere if they conflict with workplace expectations or sociocultural values [61,76]. For instance, physical activity during work hours may be seen as unprofessional despite its proven benefits for musculoskeletal and mental health [61,76]. Economic disparities and limited infrastructure also constrain access to ergonomic equipment and wellness programs, particularly in low-resource settings [48,49]. Even with awareness, cultural beliefs about health, discipline, and professional identity shape adoption of healthy behaviors [61,76,77,78,79]. To address these challenges, intervention models should be adaptive rather than prescriptive, combining affordable ergonomic adjustments, culturally relevant education, peer-led support, and scalable digital tools [90]. Culturally tailored designs, both linguistically and socially, enhance adoption and adherence [91]. Co-creating interventions with local stakeholders, maintaining flexibility, and respecting cultural practices increases program success and promotes sustainable, health-positive institutional change [61,62,76,107,108].

### 4.4. Long-Term Sustainability of Wellness Practices

Sustaining wellness requires institutional structures that ensure continuity, accountability, and engagement beyond individual motivation [67,79]. Simply introducing physical activity or ergonomic measures is insufficient without reinforcement through performance metrics, incentives, and designated wellness champions [91]. Sustainability studies highlight the value of gamification and engagement tools, such as leaderboards, recognition systems, and personalized feedback, which enhance participation and maintain health improvements even years after implementation [92,105,106,107,111,112,113,114]. Multi-component systems that integrate environmental cues, organizational norms, and psychological support help embed wellness as a cultural shift in daily practice rather than as a standalone program [61,62,63,76]. At a broader level, intersectoral collaboration among dental associations, public health agencies, and occupational safety bodies is essential to standardize guidelines and align wellness programs with health policy, supporting workforce retention and reducing preventable costs [78,79]. Finally, ongoing interdisciplinary research in ergonomics, stress physiology, cognitive workload, and behavioral economics is critical to developing adaptive, evidence-based interventions that reflect the realities of dental practice [93,107,108,109,114].

### 4.5. Limitations and Future Directions

This review has several limitations that should be acknowledged. The heterogeneity of included studies, in terms of design, outcomes, and populations, limited the ability to synthesize findings quantitatively or establish causality. Most studies were cross-sectional, precluding longitudinal insights into the sustained impact of wellness interventions. Furthermore, much of the evidence originates from high-income countries, reducing generalizability to resource-constrained or culturally distinct settings. Future research should prioritize longitudinal and interventional designs to evaluate the long-term effectiveness and feasibility of integrated wellness strategies. Studies exploring culturally adaptive, context-sensitive interventions, as well as the role of emerging technologies in promoting occupational health, are also particularly needed. Interdisciplinary research that incorporates biological, psychological, organizational, and policy perspectives can help develop more evidence-based models to support resilience and sustainability in dental practice.

Although this review primarily focuses on the role of physical activity in preventing musculoskeletal disorders and promoting overall wellness among dental professionals, broader evidence suggests additional, biologically significant mechanisms that warrant further exploration. Within the broader literature, physical activity has been linked to reductions in senescent cell accumulation, enhanced mitochondrial function, and the preservation of telomere length, factors that may counteract the biological effects of chronic occupational stress and potentially extend health span [93]. It is also linked to improved insulin sensitivity, more balanced cortisol rhythms, and strengthened immune and neurological resilience [94]. At the cognitive level, regular exercise supports neuroplasticity through the upregulation of neurotrophic factors such as BDNF, VEGF, and IGF-1, which contribute to enhanced memory, emotional regulation, and stress resilience, capabilities that are particularly relevant for dental professionals managing high psychosocial demands [93,94,95,96,97,98,99,100]. While these pathways are well-documented in the general population, future research should investigate their specific relevance in dental practitioners to determine how these biological and neurocognitive effects of physical activity may contribute to long-term resilience, reduced burnout, and professional longevity [93,95,96].

## 5. Conclusions

This review advances the understanding of occupational health in dentistry by moving beyond previous studies that primarily quantified the prevalence of musculoskeletal disorders. It highlights the connection of musculoskeletal strain, burnout, and biological aging, particularly elevated in the post-COVID-19 context by integrating evidence on physical, psychological, and behavioral dimensions. The findings, drawn from the included studies and supported by the broader literature, report on the critical role of physical activity as a modifiable factor that improves resilience, cognitive performance, and cellular health while reducing occupational stress. Complementary strategies such as ergonomic education, sleep optimization, and institutional support emerge as essential components for sustaining long-term well-being. Finally, this review also emphasizes the importance of innovative approaches, including digital tools, culturally sensitive interventions, and leadership-driven wellness initiatives, to design and support healthier, more resilient dental workplaces. Overall, a paradigm shift toward holistic, preventive care is imperative to safeguard practitioner health and ensure high-quality patient outcomes.

## Figures and Tables

**Table 1:** Study characteristics and key findings/outcomes of included studies (*n* = 28).

No	Authors, Country, Year	Study Design	Sample and Population	Setting	Outcomes and Key Findings
1	Eminoğlu et al., Turkey, 2025 [38]	Cross-sectional	234 dentists	Clinical	Dentists reporting regular PA had significantly lower prevalence and intensity of MSDs, particularly neck and back, compared to sedentary peers.
2	Sezer & Sıddıkoğlu, Turkey, 2025 [30]	Cross-sectional	298 dental students	Academic	Clinical students had higher WMS and burnout than preclinical students, linked to workload, long clinical hours, and poor posture.
3	Al-Emara et al., Finland, 2024 [39]	Cross-sectional	255 dentists	Clinical	MSDs associated with reduced work ability, higher absenteeism and lower productivity; exercise and loupes partially mitigated effects.
4	Azimi et al., Afghanistan, 2024 [4]	Cross-sectional	206 dentists	Clinical	High fatigue, stress, and back pain; regular PA reduced both physical and psychological symptoms.
5	Matur et al., Turkey, 2023 [40]	Case–control	74 dentists and 61 office staff	Clinical	Dentists had higher CTS symptoms than office staff; risk linked to repetitive hand motions and static postures.
6	Al-Huthaifi et al., Yemen, 2023 [37]	Cross-sectional	150 dentists	Clinical	Poor ergonomic knowledge and high workload contributed to elevated MSDs, especially shoulders and lower back.
7	Macrì et al., Italy and Peru, 2023 [41]	Cross-sectional	700 dentists	Clinical	Similar MSD patterns in both countries; neck and back pain most common; cultural/systemic factors influenced severity.
8	Javed et al., Pakistan, 2023 [42]	Cross-sectional	190 dentists	Clinical	Female dentists reported higher CTS symptoms, mental distress and lower PA than males; mental health support inadequate.
9	Almeida et al., Portugal, 2023 [29]	Systematic review	19 studies (students)	--	Review: dental students had high MSD prevalence, especially cervical and lumbar, often beginning in training; posture and stress were key.
10	Chenna et al., India, 2022 [43]	Systematic review	21 studies	--	Meta-analysis: global MSD prevalence among dentists 64–93%; neck, shoulders and back most affected; PA and ergonomics recommended.
11	Daou et al., Lebanon, 2022 [44]	Experimental	300 dentists	Clinical	High MSD prevalence due to long hours and poor ergonomics; dentists recommended better training and equipment.
12	Asaduzzaman et al., Bangladesh, 2022 [36]	Cross-sectional	170 dentists	Clinical	Senior dentists reported higher MSDs and more ergonomic strain than juniors, suggesting cumulative exposure effects.
13	AlDhae, Bahrain, 2022 [45]	Cross-sectional	320 dentists	Clinical	High occupational stress and low PA rates; poor awareness of ergonomic practices contributed to widespread MSD complaints.
14	Zhou et al., China, 2021 [46]	Cross-sectional	200 dentists and office workers	Hospital	Dentists had higher neck pain intensity and reduced pain thresholds than office workers, due to static postures and precision tasks.
15	Gandolfi et al., Italy, 2021 [10]	Cross-sectional	310 dentists	Clinical	MSD prevalence higher in women and experienced dentists; PA protective against symptoms.
16	Alnaser et al., Kuwait, 2021 [47]	Cross-sectional	250 dentists	Clinical	High workload, poor posture and insufficient breaks were main predictors of MSDs; absenteeism had economic impact.
17	Berdouses et al., Greece, 2020 [48]	Cross-sectional	300 dentists	Clinical	Over 50% of dentists reported MSDs, especially lower back and shoulders; poor posture and dental noise contributed.
18	Pavičin et al., Croatia, 2020 [49]	Cross-sectional	350 dentists	Clinical	High injury rates (needlestick, back strain, eye injury) among dentists, linked to posture, noise and stress.
19	AlAbdulwahab et al., Saudi Arabia, 2020 [50]	Cross-sectional	290 dentists	Clinical	Sedentary behavior linked to MSDs and obesity; lack of PA worsened quality of life.
20	Harris et al., Canada, 2020 [51]	Cross-sectional	500 dental hygienists	Clinical	83% of hygienists experienced ≥ 1 MSD; symptoms worsened with years of practice; CTS and tendinitis most common.
21	Miron et al., Romania, 2018 [7]	Cross-sectional	180 dentists	Clinical	High psychological stress and unhealthy coping (e.g., smoking); PA associated with better emotional resilience.
22	Ahmad et al., Pakistan, 2015 [52]	Cross-sectional	150 dentists	Clinical	Female dentists reported lower PA and more mental health complaints than male colleagues.
23	Memarpour et al., Iran and UAE, 2013 [53]	Cross-sectional	210 dentists	Clinical	High burnout and stress among dentists; PA, rest breaks and improved ergonomics reduced complaints.
24	Hashim & Al-Ali, Dubai and UAE, 2013 [54]	Cross-sectional	200 dentists	Clinical	Low PA, poor diet and high smoking prevalence associated with systemic health problems and MSDs.
25	Singh & Purohit, India, 2012 [55]	Cross-sectional	150 dentists and students	Academic	Students and interns had higher PA and fewer MSDs than senior faculty; lifestyle and hierarchy influenced outcomes.
26	Ellapen et al., South Africa, 2011 [56]	Retrospective	94 dentists	Clinical	Widespread back, neck and shoulder pain attributed to poor ergonomics, high patient loads and lack of recovery.
27	Sharma & Golchha, India, 2011 [57]	Cross-sectional-Questionnaire	102 dentists	Clinical	Awareness of PA benefits linked to fewer MSD symptoms and better prevention practices.
28	Kierklo et al., Poland, 2011 [58]	Cross-sectional-Questionnaire	220 dentists	Clinical	>80% of dentists reported MSDs, especially neck and back; years of practice and lack of breaks correlated with severity.

**Table 2:** Sensitivity Analysis Summary for detailed outcomes.

Sensitivity Condition	Impact on Findings	Effect Description
Exclusion of small sample studies (<100 participants)	Low to moderate	Trends remain consistent, with minor statistical shifts
Inclusion of studies with standardized MSD diagnostics only	Minimal	Findings confirm reliability across consistent diagnostics
Exclusion of studies from LMICs	Moderate to high	Contextual insights reduced, skew toward high-income settings
Inclusion of studies specifying sector (public/private)	Significant (data reduction)	Weaker conclusions due to limited data
Inclusion of studies published after 2020 only	Shift in thematic focus (less emphasis on MSDs)	More focus on mental health; traditional MSD patterns diluted

## Data Availability

No new data were created or analyzed in this study. Data sharing is not applicable to this article.

## Supplementary Materials

The following supporting information can be downloaded at: https://www.mdpi.com/article/10.3390/healthcare13161985/s1. Table S1: PRISMA 2020 Checklist [115]. Table S2: Study appraisal with reliability status.
