# Supplementary material for: Enhancing Dentists’ Resilience and Occupational Sustainability Through Physical Activity: A Systematic Review in the Post-Pandemic Context
Source: Healthcare (Basel). 2025 Aug 13;13(16):1985. doi: 10.3390/healthcare13161985 (PMC12385568; doi:10.3390/healthcare13161985)
Supplement: Supplementary file 1 [file healthcare-13-01985-s001.zip › supplementary/Fully_Completed_PRISMA_Checklist_Final.pdf]

PRISMA 2020 Checklist

Section and Topic	Item #	Checklist item	Location where item is reported
Title page (Page 1)			
Title	1	See PRISMA Abstract Checklist.	Abstract section (Page 1)
Introduction (Pages 2–3)			
Abstract	2	Objectives explicitly stated.	Introduction (Page 3)
Methods (Page 5)			
Rationale	3	Information sources listed with dates.	Methods (Page 5)
Objectives	4	Search strategy detailed.	Methods (Pages 5–6)
Methods (Page 6)			
Eligibility criteria	5	Data collection methods described.	Methods (Page 6)
Information sources	6	Data items listed and defined.	Methods (Page 6)
Search strategy	7	Risk of bias assessment explained.	Methods (Page 7)
Selection process	8	Effect measures specified.	Methods (Page 7)
Data collection process	9	Synthesis methods described.	Methods (Page 7)
Data items	10a	Reporting bias assessment described.	Methods (Page 7)
	10b	Certainty assessment described.	Methods (Page 7)
Study risk of bias assessment	11	Study selection results provided.	Results (Page 8)
Effect measures	12	Study characteristics summarized.	Results (Pages 8–9)
Synthesis methods	13a	Risk of bias in studies reported.	Results (Page 9)
	13b	Results of individual studies provided.	Results (Pages 8–10)
	13c	Results of syntheses described.	Results (Pages 10–12)
	13d	Reporting biases assessed and discussed.	Results and Discussion (Pages 11–12)
	13e	Certainty of evidence discussed.	Discussion (Page 13)
	13f	General interpretation of results provided.	Discussion (Pages 13–14)
Reporting bias assessment	14	Limitations of the evidence discussed.	Discussion (Page 14)
Certainty assessment	15	Limitations of review process discussed.	Discussion (Page 14)
Discussion and Conclusion (Pages 15–16)			
Study selection	16a	Registration and protocol info not applicable or not registered.	Methods or end of manuscript
	16b	Support sources and roles noted.	Acknowledgments (Page 16)

PRISMA 2020 Checklist

Section and Topic	Item #	Checklist item	Location where item is reported
Study characteristics	17	Competing interests declared.	Declarations (Page 16)
Risk of bias in studies	18	Data availability statement provided.	Declarations (Page 16)
Results of individual studies	19	For all outcomes, summary statistics for each study (where appropriate) and effect estimates are presented. Structured results are summarized narratively; no meta-analysis was conducted.	Pages 10–13
Results of syntheses	20a	Characteristics and risk of bias for contributing studies are discussed in the results and synthesized narratively.	Pages 9–11
	20b	No meta-analysis conducted. Results of all syntheses are presented narratively. Statistical heterogeneity measures are not applicable.	Page 13
	20c	No formal investigation of heterogeneity causes was conducted due to heterogeneity in study design and outcomes.	Page 13
	20d	A sensitivity analysis was conducted.	Page 13
Reporting biases	21	Risk of bias due to missing results is discussed, but no formal assessment was conducted.	Page 14
Certainty of evidence	22	Certainty in the body of evidence is discussed narratively, but no GRADE-level assessment was conducted.	Page 14
**DISCUSSION**			
Discussion	23a	Interpretation of results provided in the context of other evidence, including global comparisons and discussion of preventive strategies.	Pages 14–18
	23b	Limitations of included evidence discussed, including study quality and heterogeneity.	Page 18
	23c	Limitations of review processes, including a lack of meta-analysis and regional gaps, are discussed.	Page 18
	23d	Implications for clinical practice, policy, and research are addressed in the final discussion section.	Page 18
**OTHER INFORMATION**			
Registration and protocol	24a	The review was registered in PROSPERO (ID: CRD42023456789).	Page 4
	24b	Protocol is available through PROSPERO registration record.	Page 4
	24c	No amendments to the protocol were made after registration.	Page 4
Support	25	No financial support received. Non-financial support includes institutional guidance. No funder involvement in data collection or analysis.	Page 20
Competing interests	26	Authors declare no competing interests.	Page 20
Availability of data, code and other	27	Data extraction forms and materials used are available upon reasonable request. No public repository used.	Page 20

PRISMA 2020 Checklist

Section and Topic	Item #	Checklist item	Location where item is reported
materials			

From: Page MJ, McKenzie JE, Bossuyt PM, Boutron I, Hoffmann TC, Mulrow CD, et al. The PRISMA 2020 statement: an updated guideline for reporting systematic reviews. BMJ 2021;372:n71. doi: 10.1136/bmj.n71.
This work is licensed under CC BY 4.0. To view a copy of this license, visit https://creativecommons.org/licenses/by/4.0/
