# Supplementary material for: Enhancing Dentists’ Resilience and Occupational Sustainability Through Physical Activity: A Systematic Review in the Post-Pandemic Context
Source: Healthcare (Basel). 2025 Aug 13;13(16):1985. doi: 10.3390/healthcare13161985 (PMC12385568; doi:10.3390/healthcare13161985)
Supplement: Supplementary file 1 [file healthcare-13-01985-s001.zip › supplementary/healthcare-3756467-non-published.pdf]

Table S1. Study Appraisal With Reliability Status

Authors	Country	Year	Type of Study	Tool Used	Reliability Status
1. Eminoğlu et al, 2025, [38].	Turkey	2025	Cross-sectional	Newcastle-Ottawa Scale (NOS)	Moderate to High
2.Sezer, &, Sıddıkoğlu, 2025, [30].	Turkey	2025	Cross-sectional	Newcastle-Ottawa Scale (NOS)	Moderate
3. Al-Emara et al, 2024, [39]	Finland	2024	Cross-sectional	Newcastle-Ottawa Scale (NOS)	Moderate
4. Azimi, et al., 2024 [4]	Afghanistan	2024	Cross-sectional	Newcastle-Ottawa Scale (NOS)	Low
5.Matur et al., 2023 [40]	Turkey	2023	Case-control	Newcastle-Ottawa Scale (NOS)	Moderate
6.Al-Huthaifi et al., 2023, [37]	Yemen	2023	Cross-sectional	Newcastle-Ottawa Scale (NOS)	Moderate
7.Macrì et al., 2023 [41]	Italy and Peru	2023	Cross-sectional	Newcastle-Ottawa Scale (NOS)	Moderate
8.Javed, et al., 2023 [42]	Pakistan	2023	Cross-sectional	Newcastle-Ottawa Scale (NOS)	Low
9.Almeida et al , 2023 [29].	Portugal	2023	Systematic review & meta-analysis	JBI Critical Appraisal Tool	High
10.Chenna et al 2022, India [43].	India	2022	Systematic review & meta-analysis	JBI Critical Appraisal Tool	High
11.Daou et al., 2022 [44]	Lebanon	2022	Cross-sectional	Newcastle-Ottawa Scale (NOS)	Moderate
12.Asaduzzaman et al., (2022). [36]	Bangladesh	2022	Cross-sectional	Newcastle-Ottawa Scale (NOS)	Moderate
13.Al Dhae, (2022) [45]	Bahrain	2022	Cross-sectional	Newcastle-Ottawa Scale (NOS)	Moderate
14.Zhou et al., 2021 [46]	China	2021	Comparative cross-sectional	Newcastle-Ottawa Scale (NOS)	Moderate
15.Gandolfi et al., (2021) [10]	Italy	2021	Cross-sectional	Newcastle-Ottawa Scale (NOS)	Moderate
16. Alnaser, et al., (2021) [47]	Kuwait	2021	Cross-sectional	Newcastle-Ottawa Scale (NOS)	Moderate
17.Berdouses et al., (2020) [48]	Greece	2020	Cross-sectional	Newcastle-Ottawa Scale (NOS)	High
18.Pavičin et al.[48]	Croatia	2020	Quantitative	Newcastle-Ottawa Scale (NOS)	Moderate
19.AlAbdulwahab et al., 2020 [50]	Saudi Arabia	2020	Quantitative	Newcastle-Ottawa Scale (NOS)	Low
20.Harris et al., 2020 [51]	Canada	2020	Cross-sectional	Newcastle-Ottawa Scale (NOS)	Moderate

21.Miron et al., (2018) [7]	Romania	2018	Cross-sectional	Newcastle-Ottawa Scale (NOS)	Moderate
22.Ahmad et al., 2015 [52].	Pakistan	2015	Cross-sectional	Newcastle-Ottawa Scale (NOS)	Low
23.Memarpour et al., (2013) [53]	Iran/UAE	2013	Cross-sectional	Newcastle-Ottawa Scale (NOS)	Moderate
24.Hashim & Al-Ali (2013) [54].	Dubai, Iran, UAE	2013	Cross-sectional	Newcastle-Ottawa Scale (NOS)	Low
25.Singh & Purohit (2012) [55]	India	2012	Cross-sectional	Newcastle-Ottawa Scale (NOS)	Moderate
26.Ellapen et al., (2011) [56]	South Africa	2011	Occupational retrospective	Newcastle-Ottawa Scale (NOS)	Moderate
27.Sharma & Gol-chha (2011) [57	India	2011	Survey	Newcastle-Ottawa Scale (NOS)	Low
28.Kierklo et al., (2011) [58]	Poland	2011	Survey	Newcastle-Ottawa Scale (NOS)	Moderate
